# Fusion of *Taq* DNA polymerase with single-stranded DNA binding-like protein of *Nanoarchaeum equitans*—Expression and characterization

**DOI:** 10.1371/journal.pone.0184162

**Published:** 2017-09-01

**Authors:** Marcin Olszewski, Marta Śpibida, Maciej Bilek, Beata Krawczyk

**Affiliations:** 1 Gdańsk University of Technology, Department of Molecular Biotechnology and Microbiology, ul. G. Narutowicza 11/12, Gdańsk, Poland; 2 Department of Food and Agriculture Production Engineering, University of Rzeszów, ul. Zelwerowicza 4, Rzeszów, Poland; University of Helsinki, FINLAND

## Abstract

DNA polymerases are present in all organisms and are important enzymes that synthesise DNA molecules. They are used in various fields of science, predominantly as essential components for *in vitro* DNA syntheses, known as PCR. Modern diagnostics, molecular biology and genetic engineering need DNA polymerases which demonstrate improved performance. This study was aimed at obtaining a new *Neq*SSB*-TaqS* fusion DNA polymerase from the *Taq* DNA Stoffel domain and a single-stranded DNA binding-like protein of *Nanoarchaeum equitans* in order to significantly improve the properties of DNA polymerase. The DNA coding sequence of *Taq* Stoffel DNA polymerase and the nonspecific DNA-binding protein of *Nanoarchaeum equitans* (*Neq*SSB-like protein) were fused. A novel recombinant gene was obtained which was cloned into the pET-30 Ek/LIC vector and introduced into *E*. *coli* for expression. The recombinant enzyme was purified and its enzymatic properties including DNA polymerase activity, PCR amplification rate, thermostability, processivity and resistance to inhibitors, were tested. The yield of the target protein reached approximately 18 mg/l after 24 h of the IPTG induction. The specific activity of the polymerase was 2200 U/mg. The recombinant *Neq*SSB-*Taq*S exhibited a much higher extension rate (1000 bp template in 20 s), processivity (19 nt), thermostability (half-life 35 min at 95°C) and higher tolerance to PCR inhibitors (0.3–1.25% of whole blood, 0.84–13.5 μg of lactoferrin and 4.7–150 ng of heparin) than *Taq* Stoffel DNA polymerase. Furthermore, our studies show that *Neq*SSB-*Taq*S DNA polymerase has a high level of flexibility in relation to Mg^2+^ ions (from 1 to 5 mM) and KCl or (NH_4_)_2_SO_4_ salts (more than 60 mM and 40 mM, respectively). Using *Neq*SSB-*Taq*S DNA polymerase instead of the *Taq* DNA polymerase could be a better choice in many PCR applications.

## Introduction

With advances in science, there is a growing use of thermostable DNA polymerases. Many new enzymes have been identified and described, predominantly those which were isolated from genera such as *Thermus*, *Thermococcus* and *Pyrococcus*. Depending on their origin and genetic variation, polymerases have different properties and be used for various purposes. *Taq* DNA polymerase isolated from the thermophilic eubacterium *Thermus aquaticus* [1] has revolutionized molecular biology and has become one of the most commonly used polymerases. It is the first thermostable enzyme ever used in PCR [2]. The native *Taq* DNA polymerase is isolated from *Thermus aquaticus* and its recombinant form is manufactured commercially using *E*.*coli* as a host. It has a relatively short half-life as compared to other thermostable polymerases isolated from Archaea. Studies have shown that it takes 45–50 minutes to deactivate half of polymerase molecules at 95°C and 9 minutes at 97.5°C [3]; hence, the quest for the shortest possible denaturation times to be used during amplification [4]. In 1 kb products, the amplification efficiency of *Taq* DNA polymerase is estimated at approx. 80%, with the CG content varying from 45 to 56% [5]. The amplification efficiency decreases with amplicon size increasing above 1 kb. As a result there is a requirement to engineer *Taq* DNA polymerases for enhanced processivity and improved performance, which is achieved by combining polymerases with thermostable DNA-binding proteins. It has been shown that the covalent combination of a DNA polymerase with the *Sso*7d protein of *Sulfolobus solfataricus* significantly increases its processivity [6]. *Taq* DNA polymerases are widely used in diagnostics. The amplification of clinical and/or environmental samples becomes more and more problematic. *Taq* DNA polymerases become completely inhibited when a PCR mixture contains 0.004% of blood [7]. It seems that hemoglobin and lactoferrin play an important role in the inhibition of the amplification process. BSA was found to be the most efficient amplification facilitator [8].

Research to date has shown that proteins which naturally bind to single- or double-stranded DNA in a PCR reaction mix, improve the yield of amplification and efficiency of long PCR products [9,10]. When polymerase is fused with such proteins, their functional properties improve considerably without affecting their stability or activity, [6, 11, 12]. In our study, we decided to fuse a *Nanoarchaeum equitans* protein with the N-terminal end of *Taq* Stoffel DNA polymerase.

We have recently identified a *Nanoarchaeum equitans* protein (a *Neq*SSB-like protein) which was found to naturally bind to DNA [13]. This protein has a single OB fold and constitutes a biologically active monomer which is similar to SSBs isolated from certain viruses. *Neq*SSB-like proteins are highly thermostable and have the ability to bind to any form of DNA (ssDNA, dsDNA) and, surprisingly, to mRNA, without any structure-dependent preferences. The half-life of the ssDNA binding activity at 100°C is 5 min and its melting temperature (Tm) is 100.2°C [13].

The aim of this study was to clone and overexpress an *E*. *coli* fusion protein composed of a *Taq* Stoffel DNA polymerase and a *Neq*SSB-like protein, to purify the resulting gene product, study its biochemical properties and suitability for use in PCR, and to see how it compares to a *Taq* Stoffel DNA polymerase.

## Materials and methods

### Construction of recombinant plasmids

A nucleotide sequence of the *Thermus aquaticus* gene encoding a Stoffel fragment of the *Taq* DNA polymerase was obtained from the GenBank database (accession number J04639.1). The *T*. *aquaticus* strain (ATCC25104) was used to isolate a genomic DNA which was then used as a template to amplify a *taq* Stoffel fragment gene by using the standard PCR amplification protocol with a Hypernova DNA polymerase (BLIRT SA, Gdansk, Poland). A DNA fragment of the *taq* Stoffel corresponding to nucleotides 997 to 2626 was obtained in PCR using the primers: F 5’ *AATTTTGTTTAACTTTAAGAAGGAGATATA*CATATGGCCCTGGAGGAGGCCC (forward) and R 5’ *GCAAGCTTGTCGACGGAGCTCGAATTC*GGATCCTTAatggtggtggtggtggtgCTCCTTGGCGGAGAGCCAG (reverse). The primers contained sequences which were complementary to the *taq* Stoffel gene (underlined), a sequence complementary to pET-30 Ek/LIC vector (italics), and an oligohistidine tag sequence (lowercase). A stop codon (TTA) was added to the reverse primer immediately after the oligohistidine sequence. After amplification, the PCR product (1703 bp) was mixed with the DNA of pET-30 Ek/LIC vector (Novagen, Madison, WI, USA) which was digested by *Bam*HI and *Nde*I enzymes (NEB, UK) and, following this, the mixture was used in a cloning experiment in which the OverLap Assembly kit was used (A&A Biotechnology, Poland). The *E*. *coli* TOP10 (Invitrogen, USA) cells were transformed with the help of a cloning mixture and several colonies were examined for the presence of a recombinant plasmid using a gel retardation assay and the restriction analysis.

Fusion with a *NeqSSB-like* gene on the N-terminal end of a *taq* Stoffel fragment corresponding to nucleotides 997 to 2626 was obtained in PCR with the use of the primers: F1 5’ GAGAGGCCGAT**GGAGGGGTCGACATGATC**GCCCTGGAGGAGGCCC (forward) and R1 5’ *GCAAGCTTGTCGACGGAGCTCGAATTC*GGATCCTTAatggtggtggtggtggtgCTCCTTGGCGGAGAGCCAG (reverse). The primers contained sequences complementary to the *taq* Stoffel gene (underlined), a sequence complementary to the pET-30 Ek/LIC vector (italics), a sequence for 6 amino acid linker residues (bolded) and a oligohistidine tag sequence (lowercase). The stop codon (TTA) was added to the reverse primer immediately following the oligohistidine sequence.

The DNA of pBAD/NeqSSB-likeHT plasmid [13] was used as a template for the amplification of the *NeqSSB-like* gene using the standard PCR amplification protocol. The forward primer was F2 5’ *ATTTTGTTTAACTTTAAGAAGGAGATATA*CATATGGATGAAGAGGAACTAATACAACTAATAATAGAAAAAACT (it contained a sequence which was complementary to the *NeqSSB-like* gene (underlined) and a sequence which was complementary to the pET-30 Ek/LIC vector (italics)), whilst the reverse primer was R2 5’ TCCTCCAGGGC**GATCATGTCGACCCCTCC**ATCGGCCTCTCCTTTAAAAGCTTTTA (it contained a sequence which was complementary to the *NeqSSB-like* gene (underlined) and a sequence for 6 amino acid linker residues (bolded). As a result of the PCR amplification, the following two products were obtained: a *taq* Stoffel gene (1703 bp) and a *NeqSSB-like* gene (793 bp). Following this, the PCR products were mixed with the DNA of the pET-30 Ek/LIC vector (Novagen, Madison, WI, USA) which was digested by *Bam*HI and *Nde*I enzymes (NEB, UK) and the resulting mixture was used in a cloning experiment in which the OverLap Assembly kit was used (A&A Biotechnology, Poland). The cloning scheme of the fusion *NeqSSB-TaqS* polymerase is shown in S1 Fig.

*E*. *coli* TOP10 (Invitrogen, USA) cells were transformed with the help of the cloning mixture and several colonies were examined for the presence of a recombinant plasmid using a gel retardation assay and the restriction analysis. The resulting pET30/NeqSSB-TaqS plasmid contained a complete Neq*SSB*-like sequence, a 6 amino acid linker (GGVDMI), a sequence of the *Taq* Stoffel DNA polymerase (amino acid residues from 317 to 832) and as His tag domain which enables the purification of the recombinant protein using the metal affinity chromatography.

The nucleotide sequences of the resulting recombinant plasmids, pET30/TaqS and pET30/NeqSSB-TaqS were confirmed by the DNA sequencing (Genomed, Poland).

### Expression and purification of TaqS and NeqSSB-TaqS DNA polymerases

The pET30/TaqS and pET30/NeqSSB-TaqS plasmids were transformed into the *E*. *coli* BL21 (DE3) RIL (Novagen, USA). The cells with a recombinant plasmid were grown to an OD_600_ of 0.4 in Luria-Bertani medium at 37°C, with the addition of kanamycin and chloramphenicol at a concentration of 50 μg/ml each, and were induced by IPTG at the final concentration of 1 mM for 24 h. The cells were centrifuged at 5000x*g* for 12 min and the pellets were resuspended in 20 ml of buffer A (50 mM Tris-HCl pH 9, 0.5 M NaCl and 5 mM imidazole). The samples were disintegrated five times for 45 s at 4°C, and centrifuged at 10000x*g* for 15 min. The supernatant was heat-treated at 70°C for 15 min and the denatured host proteins were removed by centrifugation. Following this, the protein was purified in a one-step process. We used the Ni^2+^-affinity chromatographic technique. The supernatant and the enzyme which was produced were put into a His•Bind Column (Novagen, USA), which was earlier prepared and equilibrated using buffer A. The recombinant proteins were washed two times using the washing buffer B (50 mM Tris-HCl pH 9, 0.5 M NaCl and 40 mM imidazole) and then eluted with the elution buffer C (50 mM Tris-HCl pH 9, 0.5 M NaCl and 300 mM imidazole). The eluted fractions were dialyzed three times against buffer D (100 mM Tris-HCl pH 8, 100 mM KCl, 0.2 mM EDTA). The trace amounts of the genomic bacterial DNA were removed using 25 U of Benzonase (Merck, Darmstadt, Germany) and MgCl_2_ at the final concentration of 5 mM. Following this, the protein sample was incubated at 37°C for 1 h. The enzyme was inactivated, by incubation at 70°C for 15 min whilst the denatured proteins were removed by centrifugation. The final formulation was prepared for storage (50 mM Tris-HCl pH 8, 50 mM KCl, 1 mM DTT, 0.1 mM EDTA, 1% Tween 20, 1% Nonidet P-40 and 50% glycerol).

### DNA polymerase activity assay

As directed in the EvaEZ Fluorometric Polymerase Activity Assay Kit Manual (Biotium, Hayward, USA), the DNA polymerase activity was assayed in an isothermal reaction at 72°C using MyGo/Pro Real-Time PCR instrument (IT-IS International Ltd., UK) in accordance with the definition of one unit of enzyme activity (“One unit of DNA polymerase activity is conventionally defined as the amount of enzyme that will incorporate 10 nmol of nucleotides during a 30-min incubation” [14]). The active DNA polymerase extended the primer to form a double-stranded product able to bind the EvaGreen dye with the resulting increase in fluorescence. The level of fluorescence was correlated with the polymerase activity and the number of bound nucleotides [14–16]. The activity was determined in relation to a commercial *Taq* DNA polymerase (Thermo Scientific, USA) with an activity of 1 U/μl.

### Optimization of PCR amplification

We optimized the working conditions for *NeqSSB-TaqS* DNA polymerases. Reactions were carried out using different buffer compositions with various pH values, which included various concentrations of MgCl_2_, KCl and (NH_4_)_2_SO_4_. In all these reactions, we used 1 mM of each dNTP, 0.4 mM of each primer, and a miniprep plasmid DNA as a PCR template with a unique known target sequence and size (PCR product of 300 bp). PCR was performed using 1U of the purified *Neq*SSB-*Taq*S DNA polymerase or *Taq*S DNA polymerase in 20 μl of the reaction mixture containing 5 ng of a DNA template. PCR was conducted as follows: an initial denaturation at 94°C for 1 min; 25 cycles of denaturation at 94°C for 15 s, annealing at 55°C for 15 s and elongation at 72°C for 15 s. After the final cycle, the sample were incubated for 5 min at 72°C.

To determine the optimum MgCl_2_ concentration, the PCR was performed at increasing concentrations of MgCl_2_ (0–9 mM) with the use of a Tris–HCl buffer. Furthermore, the PCR was carried out using various concentrations of KCl and (NH_4_)_2_SO_4_ (10–90 mM) for various pH values ranging from 7.0 to 9.0 for a Tris–HCl buffer (pH values were measured at a temperature of 25°C).

Thermostability was assayed as described by Dabrowski and Kur [17]. The purified *Neq*SSB-*Taq*S and *Taq*S DNA polymerases were heated up to 95°C and 99°C for 1, 5, 10, 20, 40 and 60 minutes.

In all our experiments, we amplified a 300 bp target fragment in a PCR using the same amount of the enzyme in the optimal conditions. We applied 10 μl of each PCR product for visualization by agarose gel electrophoresis. The relative activity of the polymerase was evaluated by densitometry with the use of GelAnalyzer 2010a program (http://www.gelanalyzer.com/). The program measured the area below the peak representing intensity of light emitted by the band on the gel. The peak of the largest field (the highest optical density) represents a 100% polymerase activity. Peaks with smaller fields (less intensive light) were compared with the largest peak and their activity was determined as a percentage of this value.

### PCR amplification rate assay

The PCR amplification rate was measured, after some modifications, using the method described by Lee et al. [11]. We used the DNA of a pET 30 plasmid containing the known target sequences as a template for the PCR in order to obtain the products with a length of 300, 500 and 1000 bp.

Amplification was performed using *Neq*SSB-*Taq*S and *Taq*S DNA polymerases in the optimal conditions for a PCR. Each PCR included the initial denaturation at 94°C for 2 min, and 25 cycles at 94°C for 15 s, at 55°C for 15 s and at 72°C for 5, 10, 15,…60 s. The PCR products were electrophoresed using the standard 1% agarose gel.

### Processivity analysis

The processivity test was carried out as described in [18], after some modifications. Eighty five μl of 20 mM Tris-HCl pH 8.3, 10 mM KCl, 10 mM (NH_4_)_2_SO_4_, 0.1% Triton X-100, 290 μM of each of the four dNTPs, 40 nM primer-template (5′-GGGGATCCTCTAGAGTCGACCTGC and 5’ TATCGGTCCATGAGACAAGCTTGCTTGCCAGCAGGTCGACTCTAGAGGATCCCC), 3 μl of EvaGreen Fluorescent DNA stain (Jena Bioscience, Jena, Germany) and 1 U of the tested polymerase were pre-incubated for 5 min at 50°C. The reactions were initiated by the simultaneous addition of 7.5 μl of 50 mM MgCl_2_ and 7.5 μl of a 0.6 mg/μl heparin trap, and the polymerization was allowed to proceed at 72°C. Aliquots (10 μl) were withdrawn after 0, 1, 2, 5, and 10 min to cool the thermoblock (4°C) and the lengths of the extended products were determined by a melting point using a MyGo/PRO Real-time PCR instrument (IT-IS International Ltd., GB). The reaction included the following stages: a pre-melt hold for 10 s at 95°C (a ramp rate of 5°C/s), the initial 60-second stage at 60°C (a ramp rate of 4°C/s) and the final 1-second stage at 97°C (a ramp rate of 0.201°C/s). The processivity was determined by comparing the melting temperature profiles of different length products serving as markers. The marker product was obtained in a PCR using the same primer as that described above and the synthetic templates which allowed the formation of products with a length exceeding the primer’s length by 1, 2, 3 through up to 20 nt.

### Primer-template binding

The binding of polymerases to the primer-template (5’-CTTCATTACACCTGCAGCTCT and 5’-CACAGCCCTGTCCCTCTTCTTC) occurred at various annealing temperatures ranging from the optimal 55°C up to 72°C, with the use of primers for the PCR amplification of a human CCR5 gene [19].

### Resistance to inhibitors

The effect of PCR inhibitors such as 0.84 μg to 54 μg of lactoferrin (Sigma-Aldrich, St. Louis, USA), 4.7 ng to 600 ng of heparin (Sigma-Aldrich, St. Louis, USA), and human blood (from a healthy volunteer) in concentrations ranging from 0.15% to 10%, on the catalytic activity of *Neq*SSB-*Taq*S and *Taq*S DNA polymerases was assessed in a PCR using the genomic DNA of *Staphylococcus aureus* as a template and primers for the specific *nuc* gene [20].

Furthermore, resistance to inhibitors present in the whole human blood was tested using primers for the amplification of human CCR5 gene [19] without the addition of any templates. PCRs were assayed as described by Kermekchiev et al. [21] with the addition of blood to the mixture (at concentrations ranging from 0.15% to 10%).

### DNA binding preferences

To demonstrate the ability of DNA polymerases to bind different types of DNA (ssDNA and dsDNA) and their preferences for binding single- or double-stranded DNA, we performed the electrophoretic mobility shift assay test of the polymerase DNA complexes. The test was performed using fluorescein-labelled oligonucleotides (dT) _76_ at the 5’ end, and a PCR product with a length of 100 bp, as described in the method outlined by Olszewski et al. 2015 [13]. The output products were analyzed using a 2% agarose gel ethidium bromide in the UV light.

## Results

### Expression and purification of TaqS and NeqSSB-TaqS DNA polymerases

The gene encoding the Stoffel fragment of a *Taq* DNA polymerase was cloned into the vector pET-30 Ek/LIC to generate a pET30/TaqS plasmid which led to the expression of the enzyme as a protein with a C-terminal polyhistidine tag. The PCR products of the *taq* Stoffel and *NeqSSB-like* genes were mixed together with the DNA of the pET-30 Ek/LIC vector in order to obtain the pET30/NeqSSB-TaqS plasmid which encodes the enzyme as a fusion protein containing the complete NeqSSB-like sequence, a 6 amino acid linker (GGVDMI) and a sequence of the *Taq* Stoffel DNA polymerase with a C-terminal polyhistidine tag. *E*. *coli* BL21 (DE3) RIL cultures with pET30/TaqS and pET30/NeqSSB-TaqS plasmids were harvested and presonicated.

Recombinant DNA polymerases were purified by passing a heat-denatured supernatant through a His•Bind Ni^2+^ affinity column. The purified *Taq*S and *Neq*SSB-*Taq*S DNA polymerases were found to have a specific activity (according to the definition proposed by Habig et al. [22]) of 1600 U/mg and 2200 U/mg respectively. These results show that the *Neq*SSB fusion did not have any negative effect on the catalytic activity of the *Taq*S DNA polymerase.

**Fig 1:**
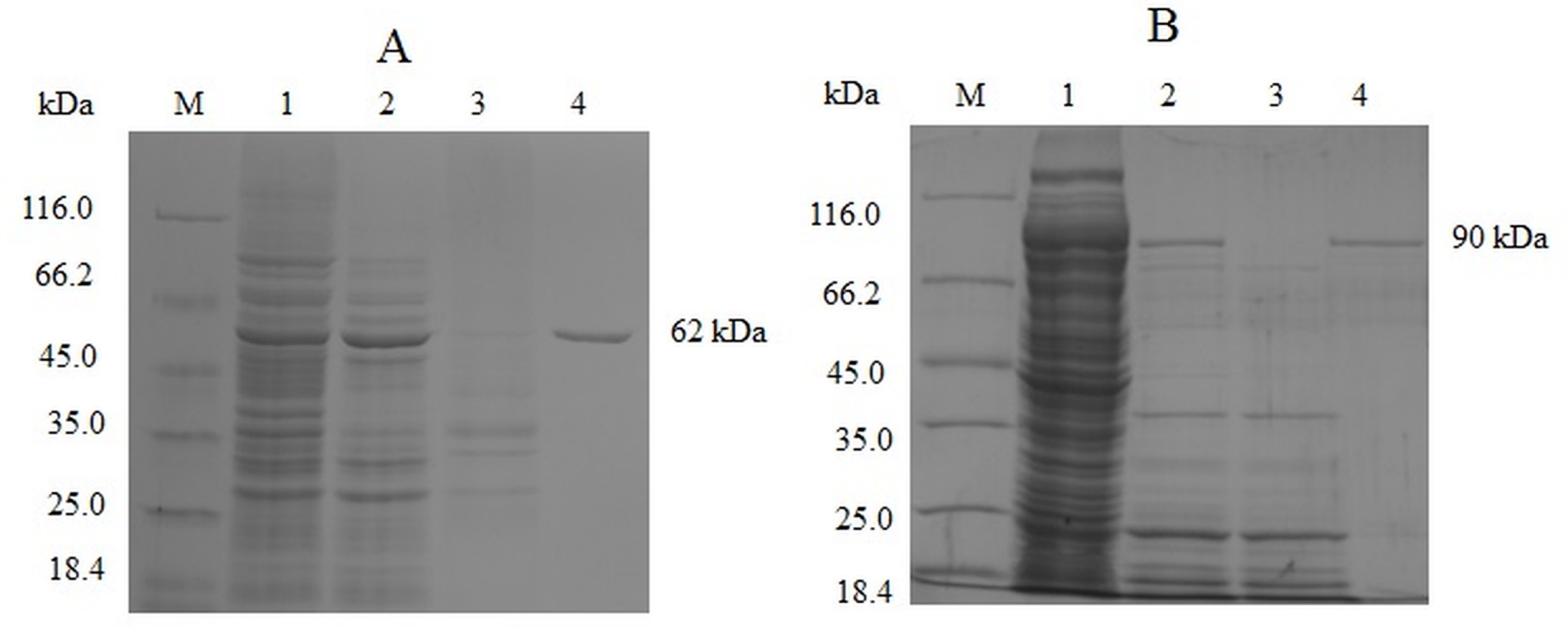
The expression and purification of *TaqS* (A) and *Neq*SSB*-TaqS* (B) DNA polymerases. The proteins were analyzed on a 10% polyacrylamide gel (SDS-PAGE). Lane M: Unstained Protein Weight Marker (Fermentas, Lithuania), molecular masses highlighted. Lane 1: the sonicated extract of induced cells; Lane 2: heat treatment; Lane 3: a by-product after the second washing with the use of the buffer B; Lane 4: purified protein after elution with the buffer C.

We recovered approximately 46% and 44% of *Taq*S and *Neq*SSB-*Taq*S DNA polymerases from the sonicated extracts, respectively. The degree of polymerase recovery and each DNA polymerase purification step were monitored with the help of SDS-polyacrylamide gel electrophoresis (Fig 1).

We observed major protein bands of 62 and 90 kDa for *Taq*S and *Neq*SSB-*Taq*S, repectively, corresponding to molecular masses of 61.8 and 89.9 kDa (the calculations were made based on the amino acid sequences). The *E*. *coli* overexpression system used in this study allowed the production of 30 mg of a *Taq*S DNA polymerase and 18 mg of a *Neq*SSB-*Taq*S fusion protein per 1 l of induced culture. Most of the native thermostable enzymes were synthesized by thermophilic bacteria at low levels and consequently they were difficult to purify [23, 24]. Another factor which contributed to such a low output was the toxicity of the overexpressed DNA-interacting proteins in the *E*. *coli* host cells. This phenomenon is common not only in DNA polymerases and SSB proteins, but also in other enzymes including restriction endonucleases [25, 26]. Several thermostable DNA polymerases in the biologically active form were produced using the same *E*. *coli* systems as those used in this study. Their expression levels in *E*. *coli* were in a range from 2.25 mg/l to 50 mg/l [27–29]. Hence, the production efficiencies of *Taq*S and *Neq*SSB-*Taq*S DNA polymerases achieved in this study were satisfactory.

### Characterization of fusion NeqSSB-TaqS DNA polymerase

The activity rather than concentration of enzymatic proteins should be compared because during the purification process the same amount of enzyme may become deactivated and then the activity will not be equivalent to concentration Hence, to obtain reliable and accurate results, this characteristic of the polymerase was assessed by the activity assay. In the subsequent experiments, the 1 U/μl activity for the *Taq*S and *Neq*SSB-*Taq*S DNA polymerases was determined by comparing it with a commercial *Taq* DNA polymerase with an activity of 1 U/μl, using a EvaEZ Fluorometric Polymerase Activity Assay Kit (Biotium, Hayward, USA), in an isothermal reaction at 72°C on a real-time PCR apparatus (IT-IS International Ltd., UK).

**Fig 2:**
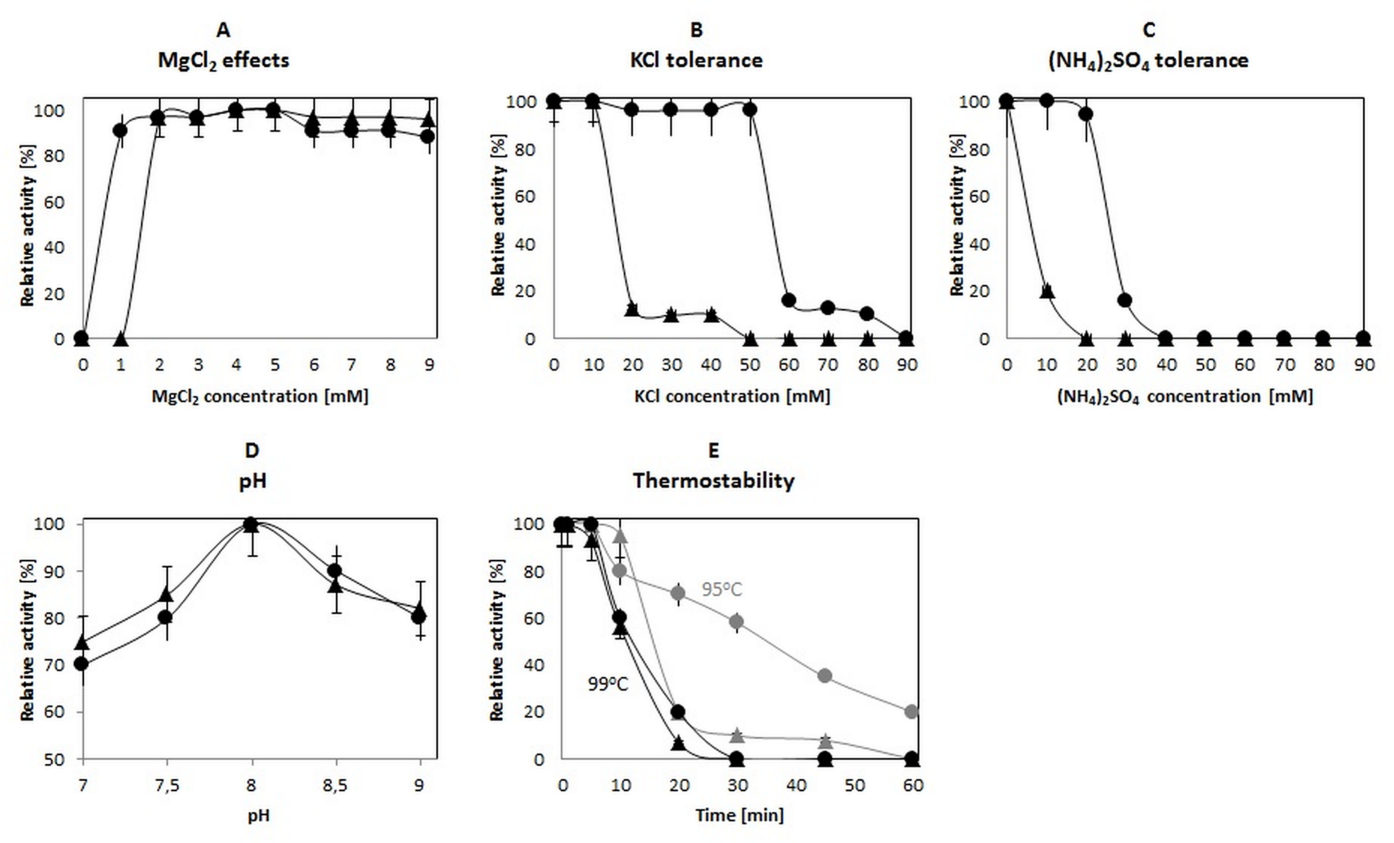
Characterization of a fusion *Neq*SSB*-TaqS* DNA polymerase in comparison to a *Taq*S DNA polymerase. The effect of (A) MgCl_2_, (B) KCl, (C) (NH_4_)_2_SO_4_, (D) pH and (E) temperature on the polymerase activity. The results for the *NeqSSB-TaqS* DNA polymerase are marked with black circles, whilst for the *Taq*S DNA polymerase with black tringles. Error bars for the *TaqS* DNA polymerase have the end bar whilst for the *Neq*-*Taq*S DNA polymerase does not have the end bar.

For characterization purposes, the polymerase activity was measured in a PCR for different buffer compositions with various concentrations of MgCl_2_, KCl or (NH_4_)_2_SO_4_ and various pHs (Fig 2). The activity of the DNA polymerase was highly dependent on MgCl_2_, whereby the maximum activities for *Taq*S DNA polymerases were within a range of 2 to 5 mM MgCl_2_ and for *Neq*SSB-*Taq*S DNA polymerases within a range of 1 to 5 mM MgCl_2_ (Fig 2A). The DNA polymerase activity was completely inhibited when KCl concentrations exceeded 20 mM for *Taq*S DNA polymerases and 60 mM for *Neq*SSB-*Taq*S DNA polymerases (Fig 2B). (NH_4_)_2_SO_4_ also had an adverse effect on *Taq*S and *Neq*SSB-*Taq*S DNA polymerase activities, inhibiting them completely when concentrations exceeded 20 and 40 mM, respectively (Fig 2C).

**Fig 3:**
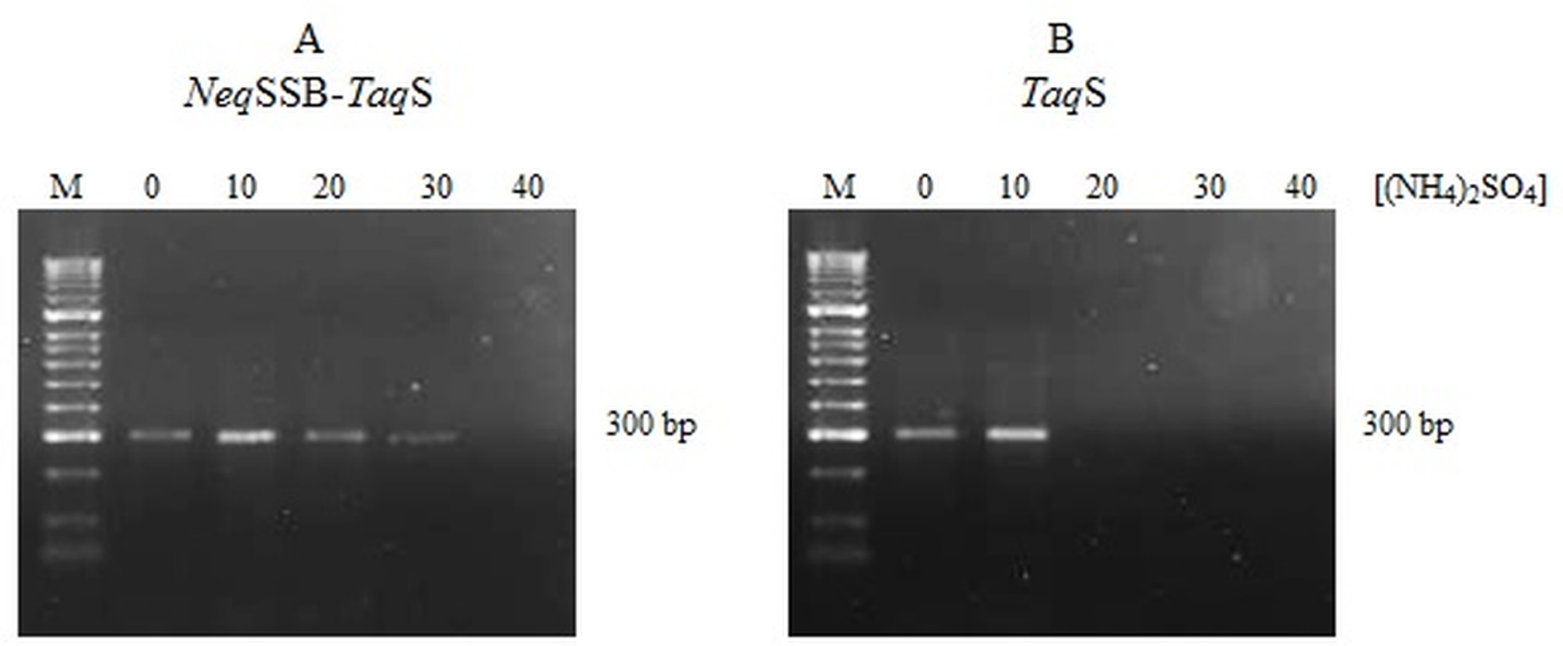
Amplification efficiency for DNA polymerases depending on the composition of salt in PCR. Differences in the amplification efficiency for the fusion *Neq*SSB*-TaqS* DNA polymerase (A) and *TaqS* DNA polymerase (B) depending on the composition of salt in the PCR buffer (10 mM KCl plus 0; 10; 20; 30; or 40 mM of (NH_4_)_2_SO_4_. Lane M: the DNA molecular size marker HyperLadder II (Bioline, UK).

The amplification efficiency for various PCR buffers depending on the composition of salt is shown in Fig 3. KCl was used at the optimum concentration of 10 mM, whilst (NH_4_)_2_SO_4_ was applied at concentrations varying from 10 to 40 mM. The positive effect of the reaction buffer at the optimum salt concentrations, 10 mM KCl and 10 mM (NH_4_)_2_SO_4_ was observed for both polymerases. However, the tolerance of the fusion *Neq*SSB-*Taq*S DNA polymerase to salt has increased. When *Neq*SSB-*Taq*S DNA polymerases were used, amplification was efficient over a broad range of KCl concentrations. These results were consistent with the results obtained for Sso7d fused with *Taq* and *Pfu* DNA polymerases which were also tolerant to high KCl concentrations [6].

The effect of pH on *Taq*S and *Neq*SSB-*Taq*S DNA polymerase activities was evaluated using buffers with a pH ranging from 7 to 9. The highest enzyme activities were observed for pH 7.5 for both polymerases (Fig 2D).

The results of the above-mentioned experiment show that the optimal buffer for *Neq*SSB-*Taq*S DNA polymerases consists of 20 mM Tris–HCl (pH 8.0), 4 mM MgCl_2_, 10 mM (NH_4_)_2_SO_4_ and 10 mM KCl.

The thermal stability of *Taq*S and *Neq*SSB-*Taq*S DNA polymerases was determined by measuring a decrease in their activity after preincubation at 95°C or 99°C. The thermal stability *of Neq*SSB-*Taq*S DNA polymerase was remarkably higher. The half-lives of *Taq*S and *Neq*SSB-*Taq*S DNA polymerases at 95°C were found to be 15 and 35 min, respectively (Fig 2E).

### PCR amplification rate and processivity

**Fig 4:**
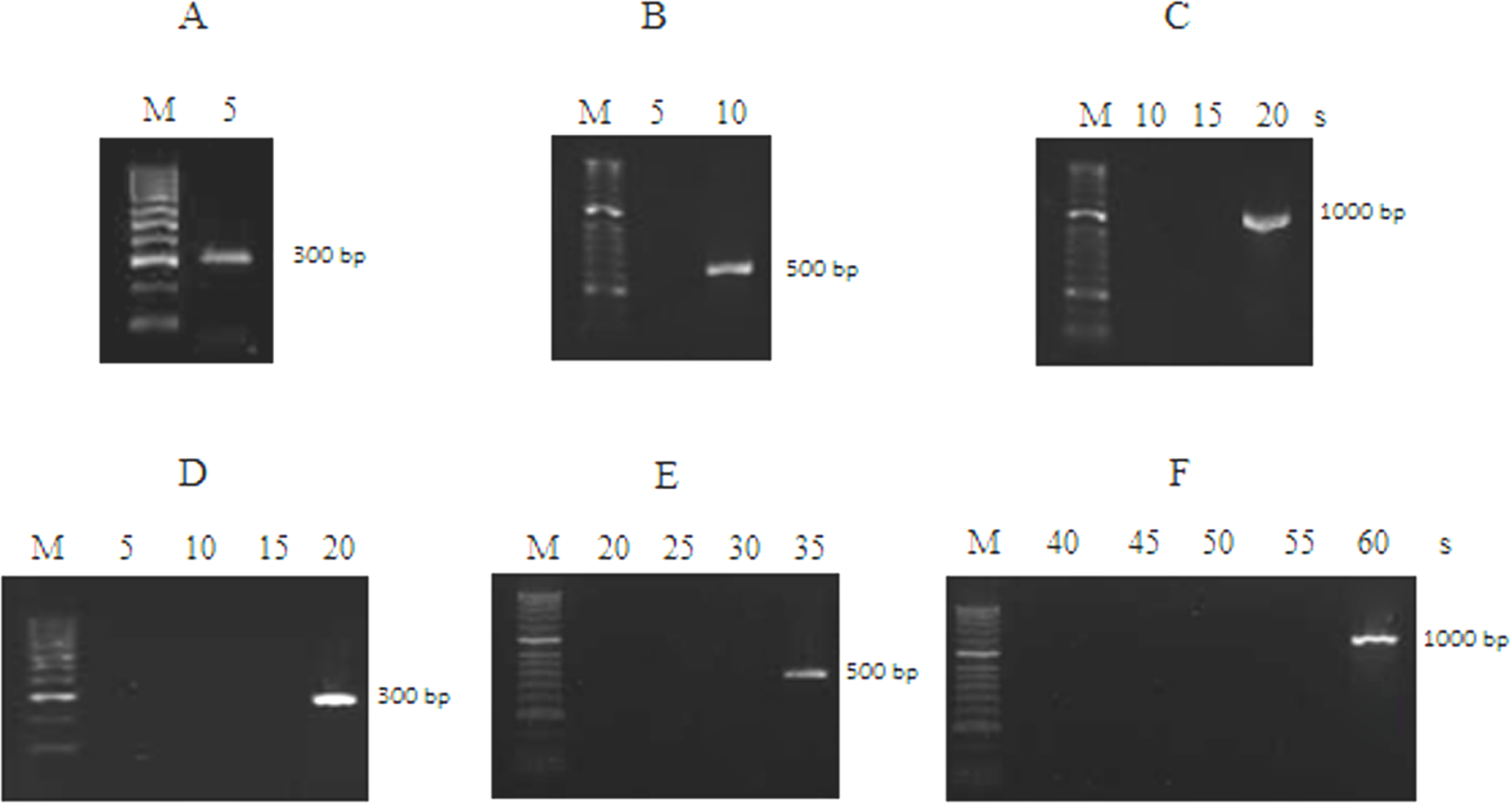
Evaluation of PCR amplification rate. Comparison of the PCR amplification rates of a fusion *Neq*SSB*-TaqS* DNA polymerase for 300 bp (A), 500 bp (B), 1000 bp (C) products and a *Taq*S DNA polymerase for 300 bp (D), 500 bp (E), 1000 bp (F) products. The elongation times used for the PCR amplification are indicated at the top. Lane M: the DNA molecular size marker (50–2000 bp).

The fusion *Neq*SSB-*Taq*S DNA polymerase replicated the template strand at a faster rate than the *Taq*S DNA polymerase (Fig 4). The *Neq*SSB-*Taq*S DNA polymerase replicated a 300 bp template within 5 s, 500 bp within 10 s and 1000 bp within 20 s, whilst the *Taq*S DNA polymerase within 20 s, 35 s and 60 s respectively. This suggests that the fusion of a *Neq*SSB protein with a *Taq*S DNA polymerase can provide a more efficient DNA amplification within a shorter reaction time.

**Fig 5:**
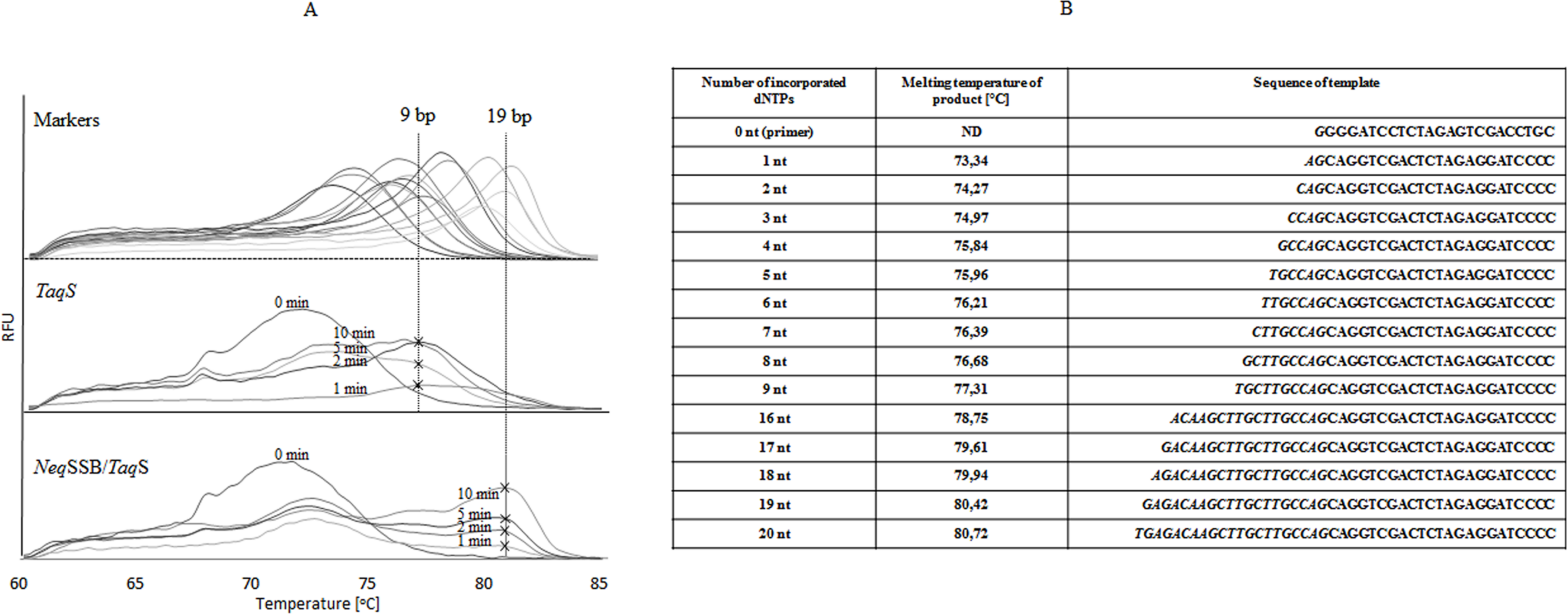
Determination of processivity based on the melting temperatures of DNA products created in the presence of a heparin trap. (A) Melting curves of the resulting products for the DNA polymerases. (B) Melting temperature of the elongated products.

The polymerase processivity is the number of dNTPs incorporated per each binding event. To ensure the correctness of measurements, polymerase molecules should associate to DNA only once and, under such single hit conditions, the processivity is expressed by the number of the incorporated dNTPs. In our experiments, heparin was used to sequester polymerase after it dissociated from the primer-template [30]. To determine processivity, the hot start conditions were created by pre-incubating the reaction mixture in the absence of Mg^2+^ and the reactions were initiated by the simultaneous addition of metal ions and a heparin trap. Following this, the processivity was determined by comparing the melting temperature profiles of products which had different known lengths and served as markers. The *Neq*SSB-*Taq*S DNA polymerase had a prominent melting peak at 80,49°C after the incorporation of 19 nucleotides, which represented processivity (Fig 5). The control *Taq*S DNA polymerase showed a lower processivity with the prominent melting peak at 77,27°C after the incorporation of 9 dNTPs (Fig 5). This suggests that the fusion of the *Taq*S DNA polymerase with the *Neq*SSB considerably improved processivity. The processivity values determined with the use of a heparin trap were much lower than the values previously published for the *Taq* DNA polymerase [31, 32] and were within a range of 80 nt to 160 nt. In these studies, no trap was used to protect against polymerase rebinding and against a repetition of the extension cycle. The differences in the results obtained may be explained by the changes in the protocol that we used.

### Primer-template binding

**Fig 6:**
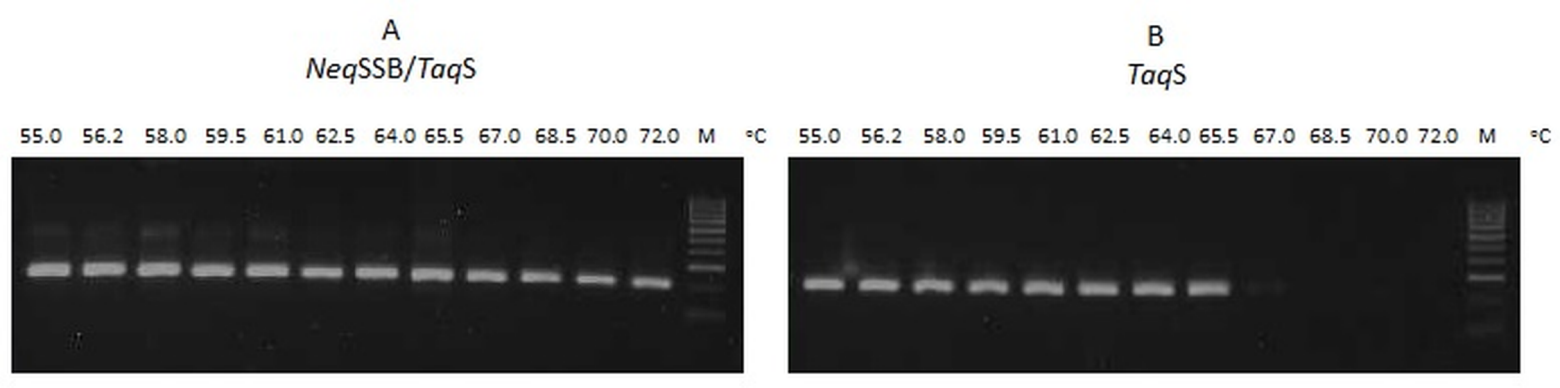
The primer-template binding for *NeqSSB-TaqS* and *TaqS* DNA polymerases. The binding of fusion *Neq*SSB*-TaqS* (A) and native *TaqS* (B) DNA polymerases to a primer-template measured at various annealing PCR temperatures (indicated in each lane at the top of the gels). The amplified products were analyzed on a 2% agarose gel stained with ethidium bromide.

The influence of the fusion of a *Neq*SSB and a *Taq*S DNA polymerase on the primer-template binding was determined using PCR reactions at various annealing temperatures ranging from the optimal temperature of 55°C up to 72°C. As shown in Fig 6, the binding affinity for the fusion polymerase increased considerably. The specific PCR product for the *Neq*SSB-*Taq*S DNA polymerase was observed at the annealing temperature of 72°C, whilst for the *Taq*S DNA polymerase at 65.5°C. This suggests that the fusion enzyme containing DNA which binds to *Neq*SSB protein, probably creates a much stronger bond to the primer-template than that created by the *Taq*S DNA polymerase.

### Tolerance of the fusion NeqSSB-TaqS DNA polymerase to PCR inhibitors

**Fig 7:**
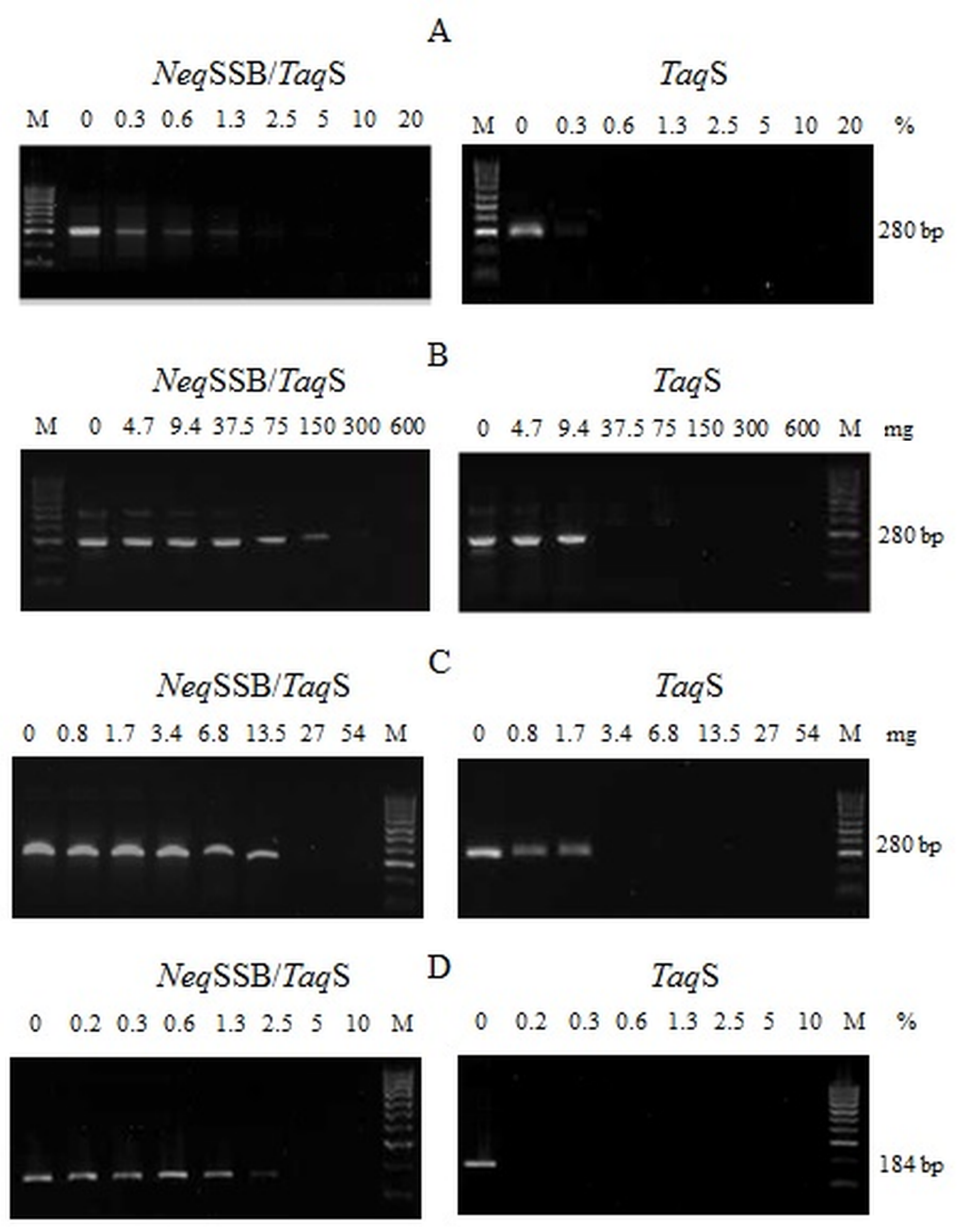
DNA polymerase tolerance to PCR inhibitors. The effect of blood (A), lactoferrin (B) and heparin (C) inhibitors on DNA amplification with the use of the genomic DNA of *S*.*aureus* as a template and primers for specific *nuc* gene detection. The effect of whole human blood on the DNA amplification with the use of primers for the amplification of a human CCR5 gene (D). No inhibitors were used in control reactions. Lane M: DNA standards ladder (100–1000 bp). The amplified products were analyzed on a 2% agarose gel stained with ethidium bromide.

The fusion *Neq*SSB*-TaqS* enzyme and the *Taq*S DNA polymerase were PCR-tested in the presence of serial dilutions of whole human blood, lactoferrin and heparin which all have been reported to inhibit PCR [7, 8, 21, 33,34]. It was shown that the fusion *Neq*SSB-*Taq*S DNA polymerase was much more resistant to all the tested inhibitors than the *Taq*S Stoffel enzyme was. *Neq*SSB-*Taq*S and *Taq*S polymerases remained functional in the presence of 0.3–1.25% and less than 0.3% of whole blood, respectively (Fig 7A), 0.84–13.5 μg and 1.68 ng of lactoferrin, respectively (Fig 7B), and 4.7–150 ng and 9.4 ng of heparin, respectively (Fig 7C).

Resistance to inhibitors including whole human blood was also tested in a PCR in which primers were used to amplify a human CCR5 gene without the addition of any template (the template came directly from the blood). As shown in Fig 7D, the fusion *Neq*SSB-*Taq*S DNA polymerase amplified the human gene target in blood concentrations of 2.5%, whilst the *Taq*S DNA polymerase was strongly inhibited at any tested blood concentration.

### DNA binding preferences

**Fig 8:**
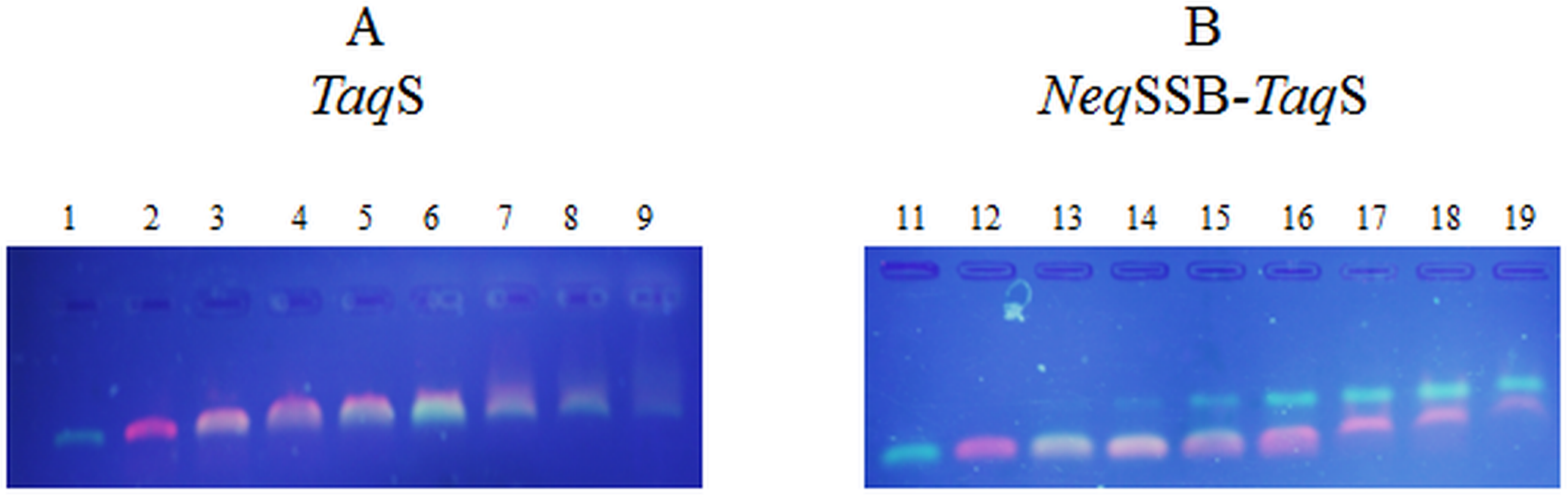
A mobility shift assay for *TaqS* DNA polymerase (A) and *NeqSSB-TaqS* DNA polymerase (B) with ssDNA and dsDNA. The output products were analyzed on a 2% agarose gel with ethidium bromide in the UV light. The reaction mix contained 10 pmol Oligo (dT)_76_ and/or 2.5 pmol PCR product with a length of 100 bp. In panel A: 1. Oligo (dT)_76_ and 0 pmol *TaqS* DNA polymerase; 2. 100 bp PCR product and 0 pmol DNA polymerase; 3–9. Oligo (dT)_76_ and 100 bp PCR product with 24,6; 49,2; 98,4; 196,8; 393,6; 787,2; 1574,4 pmol of *TaqS* DNA polymerase, respectively. In panel B: 11. Oligo (dT)_76_ and 0 pmol *NeqSSB-TaqS* DNA polymerase. 12. 100 bp PCR product and 0 pmol *NeqSSB-TaqS* DNA polymerase. 13–19. Oligo (dT)_76_ and 100 bp PCR product with 3,3; 6,6; 13,2; 26,4; 52,8; 105,6; 211,2 pmol *Neq*SSB-*Taq*S DNA polymerase, respectively.

The preference test in the form which was proposed by Olszewski et al. 2015 [13] allowed us to demonstrate the DNA polymerase’s ability to bind single or double stranded DNA. Furthermore, the test indicated the DNA polymerase’s preferences for the particular types of DNA: ssDNA is represented by oligonucleotides (dT)_76_ (green shine), whereas dsDNA is represented as a PCR product (100bp) (pink shine). The results of gel electrophoresis of enzyme-DNA complexes are shown in Fig 8A and 8B.

The results indicate that the *NeqSSB-TaqS* fusion DNA polymerase has a greater ability to bind both with ssDNA and dsDNA. Partial ssDNA binding is already seen at 3.3 pmol of the *NeqSSB-TaqS* DNA polymerase (Fig 8B, lane 13), whilst the full ssDNA binding occurs at 26.4 pmol (Fig 8B, lane 16). The binding of dsDNA is observed at 52.8 pmol of the fusion DNA polymerase (Fig 8B, lane 17).

In the case of the *TaqS* DNA polymerase, an interaction with DNA starts to be observable when it has a 7.5-fold higher concentration than the fusion DNA polymerase (Fig 8A). The *TaqS* DNA polymerase has been shown to have partial ability to bind to both single and double stranded DNA at a concentration of 24.6. pmol, without clearly indicated preference. The *NeqSSB-TaqS* fusion DNA polymerase shows a noticeable preference for ssDNA over dsDNA. Furthermore, the results indicate that polymerases interact with DNA in a completely different way. The fusion polymerase creates distinct complexes typical of the interaction between SSB protein and DNA [13]. Such complexes do not occur for *TaqS DNA* polymerase. These observations suggest that if the *NeqSSB-TaqS* polymerase contains NeqSSB protein then it creates a significantly higher level of affinity between the *NeqSSB-TaqS* fusion polymerase and ssDNA.

## Discussion

Currently, the PCR method is in common use in diagnostics, molecular biology and genetic engineering. Amplification efficiency is strongly dependent on DNA polymerase and reaction conditions. Modern diagnostic methods and genetic engineering techniques require the use of new types of DNA polymerases with better properties as regards higher processivity and/or amplification rates.

Available research suggests that the binding of enzymes to the template DNA is a very important stage in the polymerization process. It induces a conformational change in the thumb subdomain, creating a close fit with a DNA molecule. Another conformation change occurs inside the enzyme during the binding of dNTP.

It has a bearing on the formation of a "closed" conformation, rotating the structural components inside the fingers subdomain towards the 3' end [35, 36]. By improving any of the above-mentioned steps we can strongly influence the final performance of the whole process. For this reason, we modify the reaction conditions for the known DNA polymerases to facilitate the first step which involves the binding of a polymerase to a DNA strand. Such modifications include the addition of SSB or PriB proteins [9, 13, 37–40]. A better example of such a modification is the creation of fusion DNA polymerases which include proteins that bind naturally to single and double stranded DNA. In our studies, to achieve proper fusion, we used the *Neq*SSB protein of *Nanoarchaeum equitans*, which binds both types of DNA. The *Neq*SSB is a small protein which has the unique properties of a hyperthermophilic protein and is active as a monomer; therefore it can be used in PCRs at denaturation temperatures exceeding 90°C. We fused the polymerase with DNA binding proteins on the N-end using 6 amino acid linker (Gly-Val-Asp-Met-Ile) in a similar way to that used in the fusion polymerase patented in 2013 [41]. The linker creates a more flexible and ‘relaxed’ fusion polymerase. Steric hindrances are avoided which may be crucial for the stable association of the fusion polymerase with the DNA template which is essential for the polymerisation process. Some studies [6, 11, 12] and our tests have shown that the covalent linking of a DNA binding protein to a DNA polymerase can strongly enhance the polymerase processivity. We have shown that a fusion polymerase which included the *Neq*SSB protein increased the elongation rate threefold and improved processivity from 9 nt to 19 nt. Inhibitors present in the tested material or which are a residue from the process of isolation of nucleic acid are the most common problem encountered during the amplification of environmental and blood samples [42]. PCR inhibitors can interact with DNA or block enzymes by degrading the DNA polymerase, by inhibiting its active centre or by blocking access to this centre for cofactors such as magnesium ions [8, 43]. Inhibitors either reduce the efficiency of PCR or block it completely. The available commercial native polymerases are not always able to deal with these problems in a PCR. Our study shows that a fusion *Neq*SSB*-TaqS* DNA polymerase created by joining a *Taq* Stoffel DNA polymerase with the *Neq*SSB-like protein of *Nanoarchaeum equitans* exhibits a much higher tolerance to PCR inhibitors (blood, lactoferrin, heparin) as compared to *Taq* Stoffel DNA polymerases. For example, the *TaqS* DNA polymerase in a PCR mixture was completely inhibited in all of the tested whole blood concentrations and the endogenous CCR5 gene did not amplify at all, unlike the *Neq*SSB*-TaqS* DNA polymerase which was able to amplify in 2.5% whole blood. High-thermostable DNA polymerases have a broad range of applications in molecular biology, especially in the amplification of GC-rich templates [43]. We showed that thermostability was remarkably higher for the *Neq*SSB-*Taq*S DNA polymerase. The half-life at 95°C was two times longer for *Neq*SSB-*Taq*S than for *Taq*S DNA polymerases. Mg^2+^ ions are a critical parameter in PCRs and the optimization of their concentration is essential for the native DNA polymerase. Unlike the *Taq*S DNA polymerase, the *NeqSSB-TaqS* DNA polymerase exhibited acceptable amplification efficiency within a wide range of Mg^2+^ concentrations (from 1 to 5 mM MgCl_2_). Furthermore, the *Neq*SSB-*Taq*S DNA polymerase had a satisfactory amplification efficiency for (NH_4_)_2_SO_4_ and KCl concentrations which were 2 and 3 times greater, respectively. The above-mentioned results are consistent with the results obtained in another study in which a fusion DNA polymerase was tested [6].

The improvement in the properties of the *NeqSSB-TaqS* fusion DNA polymerase results from a much better affinity to the DNA template and is also due to different binding mechanisms as indicated in the results of the DNA binding assay.

The presence of an additional SSB protein which has a natural binding affinity to both ssDNA and dsDNA increases the polymerase affinity for both types of the nucleic acids. Research indicates that the natural binding affinity between the *TaqS* polymerase and dsDNA, and especially ssDNA, is significantly lower than that for the *TaqS* polymerase in fusion with NeqSSB. The *TaqS* polymerase started to interact with DNA in a noticeable but very limited manner at protein concentrations which were at a level at which the fusion polymerase was already able to completely bind to both single- and double-stranded DNA.

This has also been confirmed by DNA binding experiments in which DNA binding proteins, eg.SSB indicated a higher affinity for DNA than for *Taq* and *TaqS* DNA polymerases [44, 45]. Furthermore, the binding of DNA by a *TaqS* polymerase occurs in a completely different way than the binding of DNA by a fusion polymerase. In the fusion polymerase, the *NeqSSB* protein is responsible for such a strong and preferential DNA binding as was shown in the case of protein-DNA complexes typical of SSB proteins [13].

The results of the binding assay enable us to better understand the results discussed in the section entitled *Primer-template binding*. The results indicate that, compared to *TaqS* reference polymerase, the *NeqSSB-TaqS* fusion polymerase is better able to stabilize the double-stranded DNA at the moment of the primer hybridization, and a PCR reaction may take place at a higher primer attachment temperature. Such a stabilization of the double-stranded structure in the presence of the *NeqSSB-TaqS* fusion polymerase can increase PCR specificity.

## Conclusions

To summarize, a fusion *Neq*SSB-*Taq*S DNA polymerase consisting of the *Taq* Stoffel DNA polymerase and the *Neq*SSB-like protein of *Nanoarchaeum equitans* exhibits a much higher extension rate, offers higher processivity and has a higher tolerance to PCR inhibitors as compared to the *Taq* Stoffel DNA polymerase. For this reason, in many PCR applications, it may be better to use a *Neq*SSB-*Taq*S DNA polymerase instead of a *Taq* DNA polymerase.

## Supporting information

S1 Fig The outline of the cloning method used to obtain a fusion *Neq*SSB*-TaqS* DNA polymerase created by joining a *Taq* Stoffel DNA polymerase and the *Neq*SSB-like protein of *Nanoarchaeum equitans*. A DNA insert for cloning was prepared using two independent PCR reactions. The amplicon obtained in **PCR1** contained the nucleotide sequence of NeqSSB, a linker coding sequence, an extra sequence complementary to the N-end of TaqStoffel and to the pET30 EK/LIC plasmid.
The **PCR2** amplicon contained the nucleotide sequence of *Taq*Stoffel DNA polymerase, a linker coding sequence and an extra sequence complementary to the C-end of *Neq*SSB and to the pET30 EK/LIC plasmid.
These products and **pET30EK/LIc** plasmid which were digested by *Nde*I and *BamH*I restriction enzymes, was used as a matrix in the Gibson reaction (OverLap Assembly kit).
(TIF)
