# Supplementary figures and images for: Fusion of Taq DNA polymerase with single-stranded DNA binding-like protein of Nanoarchaeum equitans—Expression and characterization
Source: PLoS One. 2017 Sep 1;12(9):e0184162. doi: 10.1371/journal.pone.0184162 (PMC5581180; doi:10.1371/journal.pone.0184162)

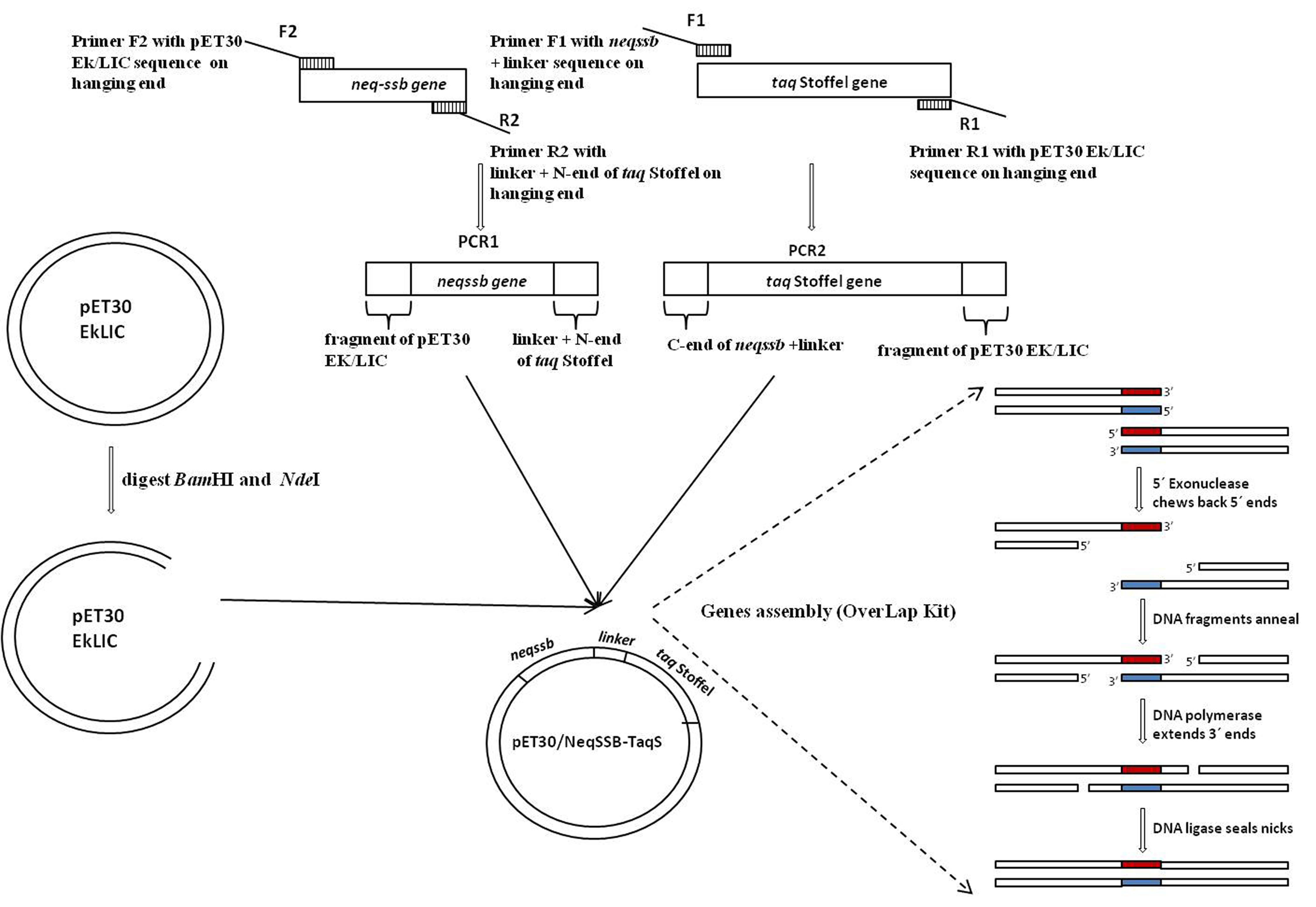

Supplement: S1 Fig — A DNA insert for cloning was prepared using two independent PCR reactions. The amplicon obtained in PCR1 contained the nucleotide sequence of NeqSSB, a linker coding sequence, an extra sequence complementary to the N-end of TaqStoffel and to the pET30 EK/LIC plasmid. The PCR2 amplicon contained the nucleotide sequence of TaqStoffel DNA polymerase, a linker coding sequence and an extra sequence complementary to the C-end of NeqSSB and to the pET30 EK/LIC plasmid. These products and pET30EK/LIc plasmid which were digested by NdeI and BamHI restriction enzymes, was used as a matrix in the Gibson reaction (OverLap Assembly kit). (TIF) [file pone.0184162.s001.tif]
